# Colistin use in a carbapenem-resistant *Enterobacterales* outbreak at a South African neonatal unit

**DOI:** 10.4102/sajid.v38i1.487

**Published:** 2023-01-30

**Authors:** Ilhaam Abrahams, Angela Dramowski, Kedisaletse Moloto, Lizel Lloyd, Andrew Whitelaw, Adrie Bekker

**Affiliations:** 1 Department of Paediatrics and Child Health, Faculty of Medicine and Health Sciences, Stellenbosch University, Cape Town, South Africa; 2 Division of Medical Microbiology, Department of Pathology, Faculty of Medicine and Health Sciences, Stellenbosch University, Cape Town, South Africa; 3 National Health Laboratory Service, Tygerberg Hospital, Cape Town, South Africa

**Keywords:** Carbapenem-resistant *Enterobacterales*, antibiotic resistance, neonate, colistin, safety

## Abstract

**Background:**

Colistin is increasingly prescribed for neonates with carbapenem-resistant *Enterobacterales* (CRE) infections.

**Objectives:**

We described patient demographics, infection episodes, treatment and clinical outcomes, colistin related adverse events and relatedness of isolates in neonates with clinically confirmed or clinically suspected CRE infections.

**Method:**

The authors retrospectively reviewed culture-confirmed and clinically suspected culture-negative CRE infections at a South African neonatal unit during a CRE outbreak.

**Results:**

Fifty-three neonates (median gestational age 29 weeks and birth weight 1185 g) were included. Twenty-three of 53 neonates (43%) had culture-confirmed CRE (17 received colistin; 6 died without receiving colistin) and 30 (57%) received colistin for clinically suspected CRE infection but were ultimately culture-negative. Prior respiratory support and surgical conditions were present in 37/53 (70%) and 19/53 (36%) neonates, respectively. Crude mortality was high (20/53; 38%) with no significant difference between culture-confirmed CRE versus clinically suspected culture-negative CRE groups (10/23 [44%] vs 10/30 [33%]; *p* = 0.45). Hypomagnesaemia (10/38; 26%) and hypokalaemia (15/38; 40%) were frequent; acute kidney injury was rare (1/44; 2%). Three CRE infection clusters were identified by genotypic analysis of 20 available isolates (18 [90%] *bla*_NDM-1_ [New Delhi metallo-beta-lactamase], 2 [10%] *bla*_OXA_ [oxacillinase]-48).

**Conclusion:**

Neonates receiving colistin therapy were predominantly preterm, with multiple risk factors for infection. Colistin-associated electrolyte derangement was frequent. Over one-third of neonates died. *Bla*_NDM-1_ was the most frequent carbapenemase gene identified in the outbreak isolates.

**Contribution:**

Colistin was safely used during an *Enterobacterales* outbreak in predominantly premature and surgical neonates. The mortality was high.

## Introduction

In 2019, 2.4 million newborns died worldwide within 28 days of birth.^1^ Neonatal bacterial infection is responsible for at least one-third of these deaths, with antimicrobial-resistant (AMR) infections estimated to contribute to 214 000 neonatal deaths annually.^2,3^ Antimicrobial resistance is a recognised global health priority, particularly in low- and middle-income countries (LMICs) neonatal units.^3,4,5,6^

The emergence of pathogens producing extended spectrum beta-lactamase (ESBL) enzymes has driven widespread carbapenem use for treating healthcare-associated infections (HAIs).^4^ As access to and use of carbapenems increased, the prevalence of carbapenem-resistant *Enterobacterales* (CRE) isolates has risen, particularly in LMICs.^4,5,6^ Carbapenemases are specific groups of β-lactamases which hydrolyse carbapenems. The three most common epidemiologically and clinically significant carbapenemases are the New Delhi metallo-beta-lactamase (NDM), specifically the NDM-1 variant predominant in Asia and the West Pacific; the *Klebsiella pneumoniae* carbapenemase (KPC) genotype, predominant in Europe and the United States (US); and the oxacillinase group (OXA), specifically the OXA-48-like enzymes.^4^

Preterm neonates, particularly those with underlying surgical conditions, those in to neonatal intensive care units (NICUs) and those with a history of prior antibiotic use, indwelling devices and prolonged hospitalisation, are at high risk of AMR infections, including CRE bloodstream infections (BSIs).^4,6,7,8^ There are limited data on the prevalence of CRE infections in African neonatal units. A tertiary care neonatal unit in Johannesburg, South Africa, reported an 8.9% CRE prevalence in BSI isolates in 2015.^7^

Most LMICs have few therapeutic options for neonates with culture-confirmed and clinically suspected CRE infections. The most widely used treatment is colistin, a polymyxin antibiotic, introduced in the fifties for Gram-negative bacterial infections. After the discovery of aminoglycosides, colistin use declined, owing to its adverse effects.^9,10^ Fuelled by the emergence of AMR Gram-negative bacterial pathogens and the paucity of other antibiotic options, colistin use has increased in LMICs.^9,11^

Intravenous polymyxin E-colistimethate sodium (CMS) is administered as an inactive prodrug, then hydrolysed to active colistin.^9^ This active form disrupts bacterial cell membrane integrity.^10^ Colistin is cleared by unknown nonrenal mechanisms and undergoes extensive renal tubular reabsorption.^9,10,12^ Data on the pharmacokinetics (PK), pharmacodynamics and safety of colistin in neonates are extremely limited,^10^ with no PK data available for preterm neonates.^8,13^ A recent study suggests that colistin-related side effects may be less common than previously thought, with reversible nephrotoxicity and apnoea reported in 5.8% and 3.9% of treated neonates, respectively.^8^

To address the paucity of data from African settings, the authors characterised patient demographics, infection episode characteristics, treatment and clinical outcomes in neonates with culture-confirmed and clinically suspected neonatal CRE infections during a CRE outbreak at a large tertiary care neonatal unit in South Africa.

## Methods

### Study setting and population

Tygerberg Hospital (TBH) provides tertiary care to Cape Town’s Metro East population as well as the Northern and Eastern rural districts of the Western Cape province. Tygerberg Hospital is a public-sector teaching hospital with 1384 beds, 8000 high-risk deliveries annually and a 37% low birthweight rate.^14^ The neonatal service has 128 beds and is the second largest inpatient unit for preterm and sick newborns in South Africa. The service includes a 12-bedded NICU, providing care to approximately 500 babies annually, with an additional 2000 neonates admitted to four neonatal wards providing a predominantly high-care service.

### Study design and data sources

The authors conducted a retrospective, descriptive study of neonates who received colistin in the first year following emergence of CRE during an outbreak between 01 December 2018 and 31 December 2019, when 23 neonates had a culture-confirmed CRE infection. An outbreak was defined as three or more cases linked in time and place. The background prevalence of CRE among *Klebsiella* BSI prior to the outbreak at the institution under study was 2%.^15^ Clinically suspected but culture-negative infections were defined as conditions prompting empiric colistin therapy and included: CRE-exposed or colonised babies developing a clinically suspected infection and a poor response to empiric (noncolistin-based) therapy for HAIs. Medical, laboratory and pharmacy data sources were used. Neonates with one or more laboratory-confirmed bloodstream, meningitis or urinary tract CRE infections were identified from the National Health Laboratory Service’s (NHLS) central data warehouse electronic laboratory records. Neonates who received a prescription for colistin were retrospectively identified from the TBH electronic pharmacy management system. Clinical and demographic data were extracted from medical records. Data captured included gestational age, birthweight, comorbidities, previous NICU admission, characteristics of episodes and treatment, as well as outcomes.

### Laboratory specimen collection and analysis

Neonates with clinically suspected HAI episodes (apnoea, respiratory distress, shock, temperature and glucose instability, tachycardia, vomiting and marked abdominal distension) underwent a complete blood count with differential, a C-reactive protein (CRP) and blood culture in line with the unit’s protocol. Urine for microscopy, culture and susceptibility testing (MC&S); cerebrospinal fluid (CSF) for cell count, chemistry and MC&S; and central venous catheter tips for MC&S were taken at the discretion of the attending clinician. Rectal swabs for CRE screening were submitted from neonates admitted to the NICU, those transferred to peripheral hospitals and neonates with a history of exposure to a patient with CRE infection. For blood cultures, 1 mL of aseptically collected blood was inoculated into a BacT/Alert PF bottle (BioMerieux, Marcy l’Etoile, France). If bacterial growth was present, a Gram-stain and sub-cultures were performed. The automated Vitek II system was used for organism identification and antimicrobial susceptibility pattern testing using Clinical and Laboratory Standards Institute (CLSI) breakpoints.^16^ Minimum inhibitory concentrations (MICs) for meropenem, imipenem and ertapenem were confirmed on all suspected carbapenem-resistant isolates from clinical samples using gradient diffusion strips (Liofilchem, Roseto degli Abruzzi, Italy).

### Molecular detection of carbapenemase genes

Genomic DNA was extracted from carbapenem-resistant isolates using a crude boil–freeze method. Isolates were screened for the carbapenemase genes *bla*_VIM_, *bla*_IMP_ and *bla*_OXA-48_ in one multiplex and *bla*_GES_, *bla*_KPC_, and *bla*_NDM_ in a second multiplex polymerase chain reaction (PCR) assay.^17^

### Strain typing

Genetic relatedness of *K. pneumoniae* isolates was investigated by repetitive extragenic palindromic (REP)-PCR typing using the KAPA 2G Multiplex Mix on the Proflex PCR system.^18^ Amplification products from the REP-PCR were visualised with gel electrophoresis and analysed on BioNumerics 7.5 (Applied Maths, US). Similarity was calculated with band matching. Dice coefficient and dendrograms were produced by the unweighted pair group method with arithmetic averages (UPGMA). Isolates with a similarity coefficient of 98% or greater were considered to belong to the same strain. Those with similarity 95% – 98% were considered related while similarity < 95% was deemed unrelated.^19^

### Empiric therapy of neonatal healthcare-associated infection

Using the unit’s empiric antibiotic recommendation guideline, antibiotic therapy for suspected neonatal HAI is initiated promptly, including piperacillin–tazobactam plus amikacin, with meropenem reserved for critically ill neonates or suspected meningitis. Vancomycin may be added at the discretion of the treating clinician. Prior to the CRE outbreak, colistin was reserved for treating carbapenem-resistant *Acinetobacter baumannii* (CRAB) infections requiring authorisation from Infectious Diseases or Microbiology, and provision of laboratory results with the prescription. During the CRE outbreak, permission was obtained to prescribe colistin without a confirmed microbiology result, after consulting Infectious Diseases or Microbiology, who would inform the hospital’s antibiotic stewardship pharmacist. Empiric and targeted therapy of colistin was administered in combination with either amikacin, meropenem, imipenem, tigecycline or tobramycin to treat presumed CRE infections in the absence of a confirmatory microbiology result. Colistin was prescribed at 40 000 IU/kg per dose intravenously, 12-hourly if less than 1 week old, thereafter 8-hourly. Colistin loading doses were not administered.

### Study definitions

Standard definitions were used to stratify neonates: preterm (< 37 weeks), low birthweight (< 2500 g), very low birthweight (1000 g – 1500 g) and extremely low birthweight (< 1000 g). In-born neonates were born at TBH; out-born neonates were born elsewhere and subsequently transferred to TBH. All neonates admitted to the neonatal service during the study period were eligible for inclusion. Prior antibiotic therapy was defined as administration of one or more systemic antibiotic doses prior to the current episode. A BSI episode was defined as a blood culture (BC) yielding a pathogen, including repeat cultures isolating the same pathogen within 14 days.^20,21^ Healthcare-associated infections were defined as infection episodes occurring at ≥ 72 h of life or hospitalisation. The authors used the Centers for Disease Control and Prevention (CDC) definition of CRE.^6^ Carbapenem-resistant *Enterobacterales* infection was defined as laboratory-confirmed culture of CRE in an aseptically collected specimen from an unwell patient; CRE colonisation was defined as the culture of CRE in the absence of disease.^22^ Targeted therapy was defined as use of colistin (with another antimicrobial based on *in vitro* susceptibility results) to treat CRE infections. Hypomagnesemia and hypokalaemia were defined as serum magnesium < 0.66 mmol/L and serum potassium < 3.5 mEq/L.^23,24^ Acute kidney injury (AKI) was defined by the Neonatal Kidney Diseases: Improving Global Outcomes (KDIGO) AKI definition.^25^ Infection-related mortality was defined as death within 72 h of BC collection.^26^ Crude mortality was defined as total deaths divided by all neonates in the study population.

### Data and statistical analysis

Continuous and categorical variables were compared using the *T*-test and the chi-squared test, respectively, for normally distributed variables and the Wilcoxon rank-sum test or Kruskal–Wallis test for nonparametric data. A *p*-value of < 0.05 was considered statistically significant. Stata statistical software version 13.0 IC (StataCorp LLC, College Station, Texas, United States) was used.

### Ethical considerations

The protocol for this study was approved by Stellenbosch University Health Research Ethics Committee (ref. no. N19/09/123).

## Results

### Study population investigated for culture-confirmed and clinically suspected culture negative infection

**FIGURE 1:**
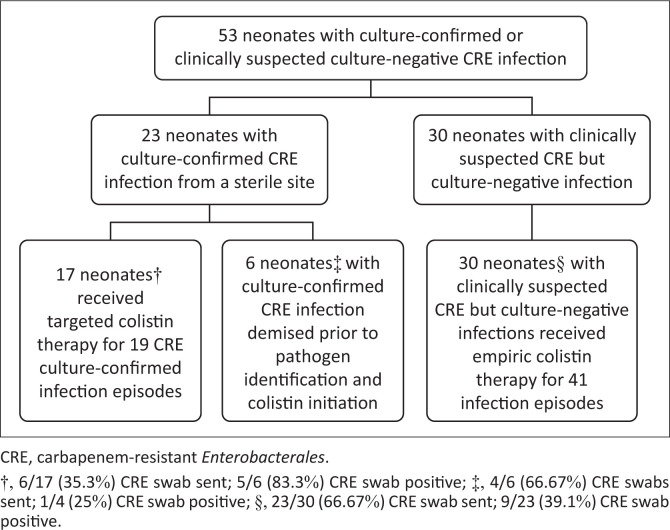
Derivation of the study population.

The authors identified 53 neonates with 66 infection episodes (Figure 1). Twenty-three of 53 neonates (43%) had culture-confirmed CRE infection and experienced 25 infection episodes. Thirty of 53 neonates (57%) were culture-negative for CRE and presented with 41 infection episodes. Within the culture-confirmed CRE infection group (*n* = 23), 17 neonates received targeted colistin therapy for 19 episodes of CRE infection; 6 neonates died very rapidly prior to culture results being available and did not receive colistin. Rectal swab CRE screening results were available for 33/53 (62.2%) neonates and nearly half (15/33; 45.4%) were CRE positive.

The median age of the cohort was 29 (interquartile range [IQR]: 28–34) weeks, and the median birthweight was 1185 (1050–1860) g (Table 1). There were no significant differences observed between the CRE culture-confirmed and the clinically suspected CRE culture-negative groups for gestational age, birthweight, gender, exposure to HIV or place of birth. There was a predominance of preterm (47/53; 88.7%) and very or extremely low birthweight neonates (35/53; 66.0%). A large proportion (37/53; 69.8%) required noninvasive ventilation for respiratory distress syndrome. Neonates in this cohort were critically ill (31/53; 58.5% were previously admitted to NICU), and most had a history of multiple prior or recent invasive procedures, including indwelling central catheters and receipt of total parenteral nutrition (TPN). Hospital stay was long, with a median duration of 51 (IQR: 31–80) days. More than one-third (19/53; 35.8%) of neonates had an underlying gastrointestinal surgical condition, two had central nervous system malformations and four had other surgical conditions including septic arthritis, laryngeal cleft, a complex cardiac lesion and choanal stenosis.

**TABLE 1:** Demographics of neonates with culture-confirmed carbapenem-resistant *Enterobacterales* infection and culture-negative infection (clinically suspected carbapenem-resistant) (*n* = 53).

Demographics	Total neonates (*n* = 53)				Culture-confirmed CRE infection (*n* = 23)				Culture-negative infection (clinically suspected CRE) (*n* = 30)				*p*
	*n*	%	Median	IQR	*n*	%	Median	IQR	*n*	%	Median	IQR	
Gestational age in weeks	-	-	29	28−34	-	-	30.1	28.3–35.0	-	-	28.5	27−32	0.123
Preterm birth (< 37 weeks)	47	88.7	-	-	20	86.9	-	-	27	90.0	-	-	0.097
Birthweight in g	-	-	1185	1050−1860	-	-	1370	1050–2110	-	-	1178	949−1770	0.323
ELBW	12	22.6	-	-	4	17.4	-	-	8	26.7	-	-	0.285
VLBW	23	43.4	-	-	9	39.1	-	-	14	46.7	-	-	0.237
LBW	13	24.5	-	-	7	30.4	-	-	6	20.0	-	-	0.369
Normal birthweight	5	9.4	-	-	3	13.0	-	-	2	6.7	-	-	0.287
Gender: male	29	54.7	-	-	14	60.8	-	-	15	50.0	-	-	0.431
HIV-exposed	14	26.4	-	-	6	26.1	-	-	8	26.7	-	-	0.962
In-born†	43	81.1	-	-	20	86.9	-	-	23	76.7	-	-	0.343
Time to onset of first infection episode (days)	12	-	-	7−23	8	-	-	7–19	19	-	-	9−30	0.004
Hyaline membrane disease requiring CPAP	37	69.8	-	-	14	60.8	-	-	23	76.7	-	-	0.145
Underlying surgical condition	19	35.8	-	-	9	39.1	-	-	10	33.3	-	-	0.663
NICU admission prior to infection episode	31	58.5	-	-	14	60.8	-	-	17	56.7	-	-	0.758
Duration of hospital stay in days	-	-	51	31–80	-	-	38	19–80	-	-	57	40−80	0.219
Crude mortality	20	37.7	-	-	10	43.5	-	-	10	33.3	-	-	0.450
Infection-attributable mortality	8	15.1	-	-	6	26.1	-	-	2	6.7	-	-	0.065

CRE, carbapenem-resistant *Enterobacterales*; IQR, interquartile range; ELBW, extremely low birthweight; VLBW, very low birthweight; LBW, low birthweight; NICU, neonatal intensive care unit; CPAP, continuous positive airway pressure.

† , In-born, born at Tygerberg Hospital.

### Characteristics of infection episodes

Infection episodes were severe, necessitating NICU admission in over half of all episodes (Table 2). Neonates presented mainly with respiratory complications, shock and temperature instability. Glucose instability (44.0% vs 17.1%; *p* = 0.017), a higher CRP (69 mg/L vs 35 mg/L; *p* = 0.015) and a lower platelet count (128 vs 260 × 10^3^ mm^3^; *p* = 0.015) were significantly more frequent in the culture-confirmed CRE group. Clinical shock (6/6; 100%; *p* < 0.001) and glucose instability (5/6; 83.3%; *p* = 0.004) were also significantly more common in neonates with culture-confirmed CRE infection who died prior to receiving colistin; these neonates also had higher CRP values (*p* = 0.051) and lower platelet counts (*p* = 0.052) than those receiving targeted or empiric colistin. Colistin therapy was significantly shorter in the empiric versus targeted colistin group (4 vs 9 days; *p* = 0.008). Among colistin-treated babies with available laboratory measurements, hypomagnesaemia (10/38; 26.3%) and hypokalaemia (15/38; 39.5%) occurred frequently. One infant in the empiric colistin group developed renal dysfunction and subsequently demised because of septicaemia and multiorgan failure.

**TABLE 2:** Clinical and laboratory features of culture-confirmed and clinically suspected culture-negative carbapenem-resistant *Enterobacterales* infection episodes (*n* = 66).

Variable	Total episodes				Targeted colistin (CRE culture-confirmed) (*n* = 23 patients)				Empiric colistin (clinically suspected CRE culture-negative) (*n* = 30 patients)				*p*
	*n*	%	Median	IQR	*n*	%	Median	IQR	*n*	%	Median	IQR	
Number of infection episodes	66	-	-	-	25	-	-	-	41	-	-	-	-
**Clinical signs**													
Increased work of breathing	39	59.1	-	-	12	48.0	-	-	27	65.9	-	-	0.152
Apnoea	21	31.8	-	-	9	36.0	-	-	12	29.3	-	-	0.569
Abdominal signs	28	42.4	-	-	9	36.0	-	-	19	46.3	-	-	0.410
Shock	19	28.8	-	-	9	36.0	-	-	10	24.4	-	-	0.312
Tachycardia	23	34.8	-	-	11	44.0	-	-	12	29.3	-	-	0.223
Temperature instability	17	25.8	-	-	9	36.0	-	-	8	19.5	-	-	0.137
Glucose instability	18	27.3	-	-	11	44.0	-	-	7	17.1	-	-	0.017
NICU admission for infection episode	-	-	35	53.1	-	-	13	52.0	-	-	22	53.7	0.896
First CRP (mg/L) value	-	-	59	18–108	-	-	69	37–164	-	-	35	6–85	0.015
Repeat CRP (mg/L) value†	-	-	59	20–121.5	-	-	70	27.5–194.5	-	-	55.5	17–112	0.132
White cell count (×10^3^ cells/mm^3^)	-	-	8.32	4.81–14.3	-	-	7.64	3.14–14.3	-	-	11.7	5.46–14.6	0.209
Haemoglobin (g/dL)	-	-	11.2	9.3–13.5	-	-	12.8	9.5–14.6	-	-	10.6	9.2–12.5	0.107
Platelet count (×10^3^ cells/mm^3^)	-	-	214	101–424	-	-	128	57–246	-	-	260	161–443.5	0.015
**Positive culture from a sterile site**													
Primary BSI	32	48.5	-	-	20	80.0	-	-	12	29.3	-	-	< 0.001
Secondary BSI	7	13.0	-	-	2‡	8.0	-	-	5§	12.2	-	-	0.701
Cerebrospinal fluid	5/37	13.5	-	-	3/16	18.8	-	-	2/21	9.5	-	-	-
Urine	4/32	12.5	-	-	3/14	21.4	-	-	1/18	5.6	-	-	-
**Total blood culture pathogen yield¶**													
*Klebsiella pneumoniae* CRE	21	31.8	-	-	21	84.0	-	-	0	0.0	-	-	-
*K. pneumoniae* ESBL	8	12.1	-	-	0	0.0	-	-	8	19.5	-	-	-
Other pathogens††	13	19.7	-	-	1‡‡	4.0	-	-	12	29.3	-	-	-
Noncolistin-based therapy at antibiotic treatment at initiation	48	90.6	-	-	22	88.0	-	-	26	63.4	-	-	-
Duration of antibiotic therapy (days)	-	-	10.0	4–15	-	-	11	4–15	-	-	9	5−15	0.705
Duration of colistin therapy (days)	-	-	5.5	2−9	-	-	9	6−20	-	-	4	2−6	0.008

CRE, carbapenem-resistant *Enterobacterales*; NICU, Neonatal Intensive Care Unit; CRP, C-reactive protein; IQR, interquartile range; BSI, bloodstream infection; ESBL, extended spectrum β-lactamase producing.

† , CRP following initiation of antibiotic therapy; secondary BSI: secondary blood stream infection;

‡ , BSI secondary to 2/2 (100%) meningitis;

§ , BSI secondary to 2/5 (40.0%); meningitis; 1/5 (20.0%) urinary tract infection (UTI); 1/5 (20.0%) central line-associated bloodstream infection (CLABSI); 1/5 (20.0%) intra-abdominal pathology;

¶ , Number of culture-positive specimens/number of specimens submitted;

†† , Other BSI pathogens cultured were three episodes of *Enterococcus faecalis* and *Staphylococcus aureus* (one MRSA), 2 of *A. baumannii*, one episode each of *Serratia marcescens, Escherichia coli, Citrobacter freundii* and *Pseudomonas aeruginosa*;

‡‡ , CRE *S. marcescens*.

The overall mortality rate was high: 20/53 (37.8%), ranging from 10/23 (43.4%) in the CRE culture-confirmed group to 10/33 (30.3%) in the empiric-therapy (CRE culture-negative) group. Of the 10 in the CRE-group who died, 6 deaths were infection-attributable and occurred prior to receiving colistin. The median time from blood culture collection to death in these six neonates was 1.5 days (IQR: 1–2), and as the blood culture result was not known at the time of death, it precluded targeted colistin therapy. In all cases, colistin therapy was initiated with other antibiotics such as amikacin, meropenem, imipenem, tigecycline and tobramycin. No colistin monotherapy was given.

### Phenotypic and genotypic analysis and treatment

Of the 66 infection episodes overall, 39/66 (59.1%) had one or more known neonatal pathogen(s) identified on BC. There were 25 CRE-infection episodes: CRE was isolated from BC in 22 episodes, and in three episodes, CRE was isolated from urine or CSF. Of the 22 CRE BSIs, CRE was also isolated from CSF and urine in one episode each. Among babies receiving empiric colistin, ESBL-producing *K. pneumoniae* was the most frequent cause of bacteraemia (7/41; 17%) (Table 2). The median time to concordant treatment was 2 days. Colistin could be stopped within 72 h of initiation in almost half of infection episodes (18/41; 43.9%) in the CRE culture-negative group, following receipt of laboratory results such as a low CRP (< 10 mg/L) or negative BC.

**FIGURE 2:**
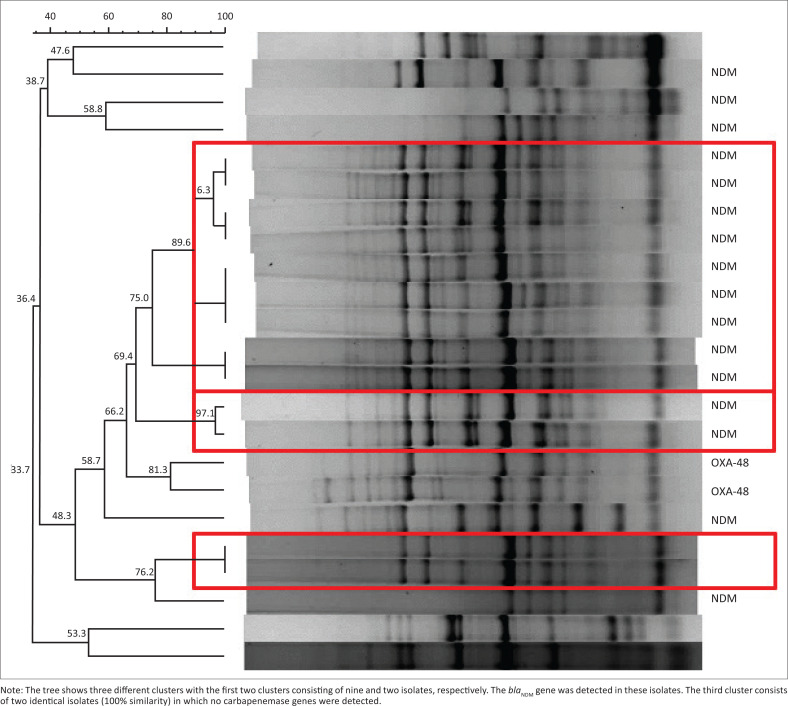
Genotypic analysis of 23 neonatal carbapenem-resistant *Enterobacterales* bloodstream infection isolates.

Polymerase chain reaction typing was available for 20 of the 23 CRE culture-confirmed cases. Only one isolate was included in the PCR typing for each infection episode. Eighteen (78.3%) had *bla*_NDM-1_ genes, and 2 (8.7%) had *bla*_OXA-48_ detected. Three clusters were found on genotyping, two of which were NDM-1 positive (Figure 2).

## Discussion

This is the first South African study describing colistin use in neonates with CRE culture-positive and CRE culture-negative infections. During a CRE outbreak in our institution 47 neonates with multiple risk factors for infection (and culture-confirmed or clinically suspected culture-negative infection episodes) received colistin therapy for 66 infection episodes. Six neonates with a CRE culture-confirmed infection died before colistin could be administered.

Patient demographics for the cohort were similar to those described in the literature: prematurity, low birthweight, previous NICU admission and the need for invasive and/or non-invasive ventilation.^7,27,28^

The only significant difference in demographics observed between neonates with a culture-confirmed infection versus those with clinically suspected CRE culture-negative infection was time to onset of infection episode (7 vs 19 days; *p* = 0.004). The median age of presentation of culture-confirmed CRE infection in this study and that of two recent South African studies was ≤ 14 days. Median age of presentation was 14 days in a Gauteng neonatal unit looking at multidrug-resistant *Enterobacterales* (MDRE), including CRE and 10.5 days in a KwaZulu-Natal study on CRE-infected neonates.^7,27^ Carbapenem-resistant *Enterobacterales* infection should therefore be considered in at-risk neonates with long hospital stays and among those with HAI episodes not responding to initial antibiotic therapy.

Presentation of CRE infection in the neonatal period is described as nonspecific with rapid progression to shock.^29^ The clinical presentation was broad. However, those with culture-positive CRE infection who demised before colistin could be initiated had significantly higher prevalence of shock and glucose instability. Patients at risk of CRE infection with these features may warrant empiric colistin treatment prior to the availability of laboratory results. The neonates in the study cohort who died also presented with elevated CRP values. Mzimela et al. similarly found that a high CRP (> 100 mg/L) in the presence of a CRE infection was associated with mortality.^27^ The numbers in both studies are, however, small.

In this study, the most common carbapenem-resistant pathogen in the CRE group was *K. pneumoniae*, while ESBL-producing *K. pneumoniae* was common in the empiric group. This is in keeping with high rates of ESBL-producing *K. pneumoniae* worldwide and the predominance of Gram-negative infections in HAI, particularly in LMICs.^8,20,22^ The *bla*_NDM-1_ was the most common carbapenemase detected in this study. In many South African hospitals, CRE has become an established pathogen, with *bla*_NDM-1_ and *bla*_OXA-48_ being the most prevalent carbapenemase types described.^7,30^

The median interval from BC collection to concordant therapy in neonates with a culture-confirmed CRE infection in this study was 2 days, while six babies with a CRE BSI demised at median time of 1.5 days from the onset of infection, before concordant therapy (colistin) could be initiated. The time from culture collection to organism identification and antimicrobial susceptibility resistance profile varies from 2 to 4 days, even in high-income countries.^31,32^ A global study looking at the concordance of empiric antibiotic therapy at initiation in 452 children (including neonates) with a BSI found the odds of mortality were increased three-fold where empiric antibiotic therapy was discordant.^32^ The rapid detection of CRE by nucleic and non-nucleic acid-based diagnostics is currently becoming more widely available or is in development and could facilitate quicker identification of resistant organisms, thus enabling more rapid initiation of targeted therapy.^33^

Acute kidney injury was detected in one neonate receiving colistin, which is consistent with low rates of nephrotoxicity described.^34^ This neonate had normal renal function prior to colistin, was on concomitant vancomycin and had multi-organ dysfunction because of sepsis, all of which potentially contributed to AKI. Possible reasons for AKI include using other nephrotoxic agents or pre-existing renal insufficiency.^35^ Electrolyte abnormalities such as hypokalaemia and hypomagnesaemia have also previously been reported in neonates on colistin therapy. Hypokalaemia was observed in 39.5% of the cohort, comparable to previous studies ranging between 16.6% and 52%.^8,36,37^ Hypomagnesaemia also occurred frequently, with 26.3% of neonates having low magnesium values, similar to the 22.0% and 23.7% reported in two studies.^38,39^

Risk factors for these electrolyte derangements in a NICU setting are multifactorial, and there may have been unaccounted confounding factors in this cohort.^23,24^ These colistin-associated adverse effects were easily managed, with no reports of treatment being stopped because of electrolyte derangements.^8,37^ Monitoring of renal function and electrolytes should be routine when receiving colistin.

The all-cause mortality related to CRE or MDRE infections in South African neonatal units ranges from 12% to 48%.^7,27,40^ In this study, the overall mortality for neonates with culture-confirmed CRE infection was 10/23 (38.5%), with all infection-related deaths occurring in patients with confirmed CRE infection that deteriorated their health very rapidly, leading to death prior to culture results being available. Delayed diagnosis and inappropriate empiric antibiotic treatment likely contributes to the high mortality observed,^29^ highlighting the need for quicker diagnostics and better guidelines on when to initiate and stop empiric colistin therapy. The use of colistin loading doses is standard practice for adult patients and is associated with improved clinical outcomes (*p* = 0.037) and microbiological clearance rates (*p* = 0.042).^41^ Pharmacokinetics data on colistin to inform dosing in neonates is limited. In 2016, a small PK study (*n* = 7) reported suboptimal plasma concentrations following administration of a single dose of colistin (150 000 IU/kg CMS), raising the question of suboptimal dosing and possible need for loading doses in this population.^13^ Conflicting data relating to mortality associated with CRE infections is likely because of varying severity of illness and timing of appropriate antibiotics,^8^ although poor culture yield and small study numbers may also play a role.^27^

Limitations of this study include the retrospective design, single centre, small sample size and unavailability of renal function and electrolytes for all neonates on colistin therapy. This study did not look at neurological side effects, apnoea or long-term follow-up. Furthermore, as therapeutic drug level monitoring was not available, it is possible that dosing of colistin was sub-optimal.

## Conclusion

The authors found colistin to be safe in this cohort neonatal with no patient requiring treatment cessation because of adverse events. Overall mortality was high in this study population. A well-functioning antimicrobial stewardship programme is reflected by the significantly shorter duration of empiric colistin versus targeted colistin in this study. With the high prevalence of CRE, guidelines are needed on when to initiate and appropriately stop colistin therapy to curtail the emergence of colistin resistance.
